# A new donor system for the patients with thalassemia: “Blood mother and blood father”

**DOI:** 10.4103/0973-6247.67036

**Published:** 2010-07

**Authors:** Duran Canatan, Ahmet Özsancak

**Affiliations:** *Department of Pediatric Hematology, Suleyman Demirel University, Isparta, Turkey*; 1 *Antalya State Hospital, Blood Center, Antalya, Turkey*

**Keywords:** Donors, thalassemia

## Abstract

**Background:**

Donor recruitment programs differ in countries depending on local conditions and causes. Regularly voluntary blood donation rate should be 5% of the population but it is extremely low in Turkey. In 1998, “Thalassemia flowers don’t fade” campaigning was started to get regular voluntary blood for patients with thalassemia. We would like to present results of our campaigning.

**Materials and Methods:**

The Thalassemia center was established in Antalya on the 1^st^ June 1994 by Ministry of Health, Turkey, because the incidence of thalassemia is very high in the Antalya region. A total of 388 patients with thalassemia were followed up regularly in the center. The annually blood requirement was approximately 5000 units per year. In 1998, a new program of blood donation for patients with thalassemia called “BLOOD MOTHER and BLOOD FATHER” was started with the support of Governor of Antalya and health management system in Antalya.

**Results:**

Between year 1998 and 2006, a total 3000 voluntary blood donors between age 18 and 65 years, of which 2160 males (72%) and 840 were females (28%), had participated in this program.

**Conclusion:**

“Blood Mother and Blood Father” campaign was successful donor recruitment program for thalassemic care. After 2006, this program is now adopted and run by Turkish Red Crescent and Thalassemia Federation of Turkey for all thalassemics in Turkey.

## Introduction

Appropriate goals of transfusion therapy and optimal safety of transfused blood are the key concepts in the protocol for routine administration of red cells to patients with thalassemia. Transfusion regimen promotes normal growth, allows normal physical activities, and adequately suppresses bone marrow activity in most patients.[1]

Thalassemia is a very important health problem in Turkey, although the prevalence rate of beta-thalassemia trait is accepted as 2% all over Turkey. In recently, Turkish National Hemoglobinopathy Council (TNHC) and Ministry of Health (MOH) have published the mean of incidence of beta-thalassemia trait was 4.3% in the Thrace, Marmara, Aegean, Mediterranean, and South Eastern regions of Turkey. The highest prevalence of the beta-thalassemia trait (13.1%) was found in the Antalya region and of the HbS trait (10%) in the Cukurova region. Hemoglobinopathy Control Program was started in 33 provinces in these regions by MOH and TNHC in 2003.[2]

With increasing life expectancy and number of thalassemic patients, the need for safe blood is increasing too, but on the other hand the numbers of donors are decreasing due to aging of donor population, infections, changing moral values, etc. And there is an important gap between demand and blood supply.[3]

Donor recruitment programs, according to each country’s specific conditions may vary with the different causes. The target of recruiting the desired number of donors will only be achieved by a properly planned recruitment strategy. One of the most important requirements for regular recruitment of blood is to achieve public instant acknowledgment. To predict future blood donation behavior and improve donor retention, it is important to understand the determinants of donor return.[4–7]

Regularly voluntary blood donation rate should be 5% of the population in any developed country but it is extremely low in Turkey. The amount of blood collected in our country is 1.5% of the population.[8]

The Blood Service Directory of Turkish Red Crescent Society (TRCS) has to organize, coordinate, evaluate, and develop blood donations all over Turkey. In fact, there are 474 Blood Centers and Blood banks in Turkey where a total of 1.400.000 units of whole blood are donated each year. This amount of blood units collected by RCS from volunteers and family donors is 60% and 40% from military personals.[9]

In 1998, “Thalassemia flowers don’t fade” campaign to find regular and voluntary blood for patients with thalassemia. On the 8^th^ of May, 2006, World Thalassemia Day, TRCS, and the Federation of Thalassemia had signed a protocol about this campaign to be followed in all blood centers. We would like to present results of this campaigning of “Blood Mother and Blood Father” between 1998 and 2006 in Antalya.

## Materials and Methods

The Thalassemia center was established in Antalya on the 1^st^ June 1994 by Ministry of Health. Because the incidence of thalassemia is very high in Antalya and surrounding region, a total of 388 patients (246 thalassemia major, 86 thalassemia intermedia, 20 sickle cell disease, 28 δ+β thalassemia, and 13 other hemoglobinopathies) were followed up regularly in the center. The annually blood requirement was approximately 5000 units per year.

In 1998, a new program of regularly blood donation for patients with thalassemia called “BLOOD MOTHER and BLOOD FATHER” was started with the support of Governor of Antalya and health management in Antalya.

Strategies of this campaign were to find enough regular and voluntary blood donors, to solve transfusion problems of thalassemic patients and contribute to their life.

Volunteers who have blood mothers and fathers do not recognize the patients ID, but they know that their blood will be given to patients with thalassemia. The “blood mothers and fathers” are pleased that they are always able to give blood to the thalassemic patients and that they can contribute to their quality of life. The patients feel close to his or her mother/father because they take blood from that person. In addition, social interaction occurs between the patients and donors.

## Results

Between years 1998 and 2006, a total 3000 persons, 2160 males (72%) and 840 females (28%), between age 18 and 65 years had participated in this program. The age distribution among blood donors were 39% in age groups 18-35 years, 48% in 36-50 years, and 13% in 51-65 years age group [Table 1].

**Table 1:** The distribution of age among blood donors

Age (years)	Number	Percentage
18-35	1170	39
36-50	1440	48
51-65	390	13
Total	3000	100

Educational status among them were 1110 primary school (37%), 480 middle school (16%), 660 high school (22%) and 750 university (25%) [Table 2].

**Table 2:** Education status among blood donors

Education status	Number	Percentage
Primary School	1110	37
Middle School	480	16
High School	660	22
University	750	25

Occupation status of them were 1050 self-employment (35%), 630 government officers (21%), 910 public workers (30.3%), 180 housewives (6%), 45 students (1.5%), 45 soldiers (1.5%), 50 retired Govt. servants (1.7 %), and 90 farmers(3%) [Table 3].

**Table 3:** Occupational status among blood donors

Occupation	Number	Percentage
Self-employment	1050	35
Governor officer	630	21
Worker	910	30.3
Housewife	180	6
Student	45	1.5
Soldier	45	1.5
Retired	50	1.7
Farmer	90	3

## Discussion

World Health Organization separates blood donors into three groups: relatives of the patient (replacement blood donors), paid donations (professional donors), and voluntary blood donors with no financial interests. Voluntary blood donation were organized by Red Crescent/Red Cross Societies, University Hospital Blood Center and the Civil Society Organizations.[3] In our country, since 1950, training, organization of blood donors regulating the private acquisition program is under the Turkish Red Crescent Society. In 1983, the Blood and Blood Products Act, authorized the State Hospital, University teaching hospitals, blood centers and donor organizations to collect the blood through voluntary blood donation camps, but this program could not be fully implemented. In 2008, the blood and blood products law was changed and blood collection was given to the Regional Blood Center of Turkish Red Crescent Society.[8,9]

No other disease other than thalassemia is connected to the hospital for lifelong, transfusion support and dependent on someone; thus keep people connected. The majority of patients require transfusions to maintain healthy life every 2-6 weeks. When they come to the hospital their colors are faded (Pallor), exhausted, groggy, and irritable. Children do not show interest to play toys; when the blood was given they became lively, and chirping, and begin to play. Children bloom like flowers after blood transfusion as if flower left in the sun after watering. Therefore, we say to these patients “Thalassemia flowers.”

In 1998, a new program of blood donation for patients with thalassemia was called “BLOOD MOTHER and BLOOD FATHER” was started in Antalya. “Blood Mother and Blood Father” campaign was successful donor recruitment program for thalassemic care. After 2006, this program is now adopted and run by Turkish Red Crescent and Thalassemia Federation of Turkey for all thalassemics in Turkey with great success.

## References

[1] (2007). Guidelines for the clinical management of thalassemia. Thalassemia International Federation.

[2] Canatan D, Kose MR, Ustundag M, Haznedaroğlu D, Ozbas S (2006). Hemoglobinopathy control program in Turkey. Community Genet.

[3] Solaz N, Bayik M, Canatan D, Politis C, Rossi U (2004). Transfuion treatment of thalassemia and other chronic anemias: Proceedings of the EST/ITSS residental course.

[4] Mayo DJ (1992). Evaluating donor recruitment strategies. Transfusion.

[5] Sandburg E, Thorton M (1994). Donor recruitment. Vox Sang.

[6] Nquven DD, Devita DA, Hirschler NV, Murpy EL (2008). Blood donor satisfaction and intention of future donation. Transfusion.

[7] Gillespie TW, Hillyer CD (2002). Blood donors and factors impacting the blood donation decision. Transfus Med Rev.

[8] Uluhan R, Berkem R, Emekdaş G, Bayik M (2008). Ulusal kan Merkezleri ve Transfüzyon Tibbi Kursu IX.03-07 Kasim.

[9] Aydinok Y, Bayik M, Canatan D, Politis C, Rossi U (2004). Report on clinical transfusion practice in thalassemia and other chronic anaemias in Turkey. Transfuion treatment of thalassemia and other chronic anemias: Proceedings of the EST/ITSS residental course.

